# Construction of an anoikis-associated lncRNA-miRNA-mRNA network reveals the prognostic role of β-elemene in non-small cell lung cancer

**DOI:** 10.1038/s41598-023-46480-7

**Published:** 2023-11-18

**Authors:** Kai Tan, Changhui Zhang, Zuomei He, Puhua Zeng

**Affiliations:** 1 Hunan University of Chinese Medicine, Changsha, 410208 Hunan People’s Republic of China; 2 Cancer Research Institute of Hunan Academy of Traditional Chinese Medicine, Changsha, 410006 Hunan People’s Republic of China; 3 Hunan Academy of Traditional Chinese Medicine Affiliated Hospital, Changsha, 410006 Hunan People’s Republic of China

**Keywords:** Cancer models, Cancer therapy, Tumour biomarkers, Non-small-cell lung cancer, Gene regulatory networks, Machine learning

## Abstract

β-Elemene is the main active ingredient in Curcumae Rhizoma that exerts antitumour effects. Anoikis affects tumour development through various biological pathways in non-small cell lung cancer (NSCLC), but the regulation between β-elemene and anoikis remains to be explored. First, we explored the molecular expression patterns of anoikis-associated genes (AAGs) using consensus clustering and characterized the impact of AAGs on patient prognosis, clinical characteristics, and genomic instability. In addition, we revealed that AAG regulatory genes have rich interactions with β-elemene targets, and established a lncRNA-miRNA-mRNA network to explain the effect of β-elemene on anoikis. Finally, to reveal the prognostic effect of their correlation, the prognostic scoring model and clinical nomogram of β-elemene and anoikis were successfully established by least absolute shrinkage and selection operator (LASSO) and random forest algorithms. This prognostic scoring model containing noncoding RNA (ncRNA) can indicate the immunotherapy and mutational landscape, providing a novel theoretical basis and direction for the study of the antitumour mechanism of β-elemene in NSCLC patients.

## Introduction

Lung cancer remains the leading cause of cancer death, accounting for 18% of all cancer deaths and 11.4% of all cancers diagnosed according to GLOBOCAN 2020, making it the deadliest type of cancer worldwide^1^. Non-small cell lung cancer (NSCLC) accounts for approximately 85% of lung cancer cases^2^. Due to the insidious symptoms of early-onset NSCLC, many patients are in advanced stages at the time of diagnosis, and thus systemic drug treatments such as chemotherapy, targeted therapy and immunotherapy are usually needed but do not provide the desired benefits due to drug resistance and adverse effects^3,4^.

Traditional Chinese medicine (TCM), especially Chinese herbal medicine (CHM), has been widely used in China and many other countries for the treatment of cancer. CHM not only relieves symptoms and improves the quality of life of cancer patients but also reduces the adverse effects and complications caused by chemotherapy, targeted therapy or immunotherapy^5^. Curcumae Rhizoma is a commonly used drug in the TCM treatment of lung cancer, and β-elemene (C_15_H_24_) is the main active ingredient extracted from it. Several previous studies have demonstrated that β-elemene plays an antitumour role in NSCLC patients not only by inhibiting NSCLC cell proliferation, invasion and migration and inducing apoptosis of NSCLC cells but also by increasing the sensitivity of drugs and other mechanisms, which are closely related to the development of NSCLC^6–8^.

Anoikis is a specific form of programmed cell death induced by the loss of cellular exposure to the extracellular matrix, which plays a key role in the maintenance of tissue homeostasis^9^. However, tumour cells have the ability to evade cell death and usually show resistance to anoikis, which leads to tumour progression and metastatic spread of cancer cells^10^. An increasing number of studies have confirmed the involvement of anoikis in NSCLC biological processes: Liu et al.^11^ showed that silencing Zic family member 2 (ZIC2) could downregulate the migration, invasion and anoikis resistance ability of NSCLC cells by inhibiting steroid receptor coactivator/focal adhesion kinase (Src/FAK) signalling; McCarroll et al.^12^ found that βIII-tubulin induced NSCLC development and anoikis resistance through the phosphatase and tensin homolog/ protein kinase B (PTEN/AKT) signalling axis; Jang et al.^13^ demonstrated that knockdown of family with sequence similarity 188 member B (FAM188B) downregulated the activity of various signalling pathways involved in anti-anoikis downstream of epidermal growth factor receptor (EGFR), sensitizing NSCLC cells to anoikis and inhibiting tumour metastasis. It is evident that anoikis resistance is regulated by multiple signalling pathways in NSCLC cells. Several studies have shown that the main pathways of β-elemene anti-NSCLC include the AMP-activated protein kinase/mitogen-activated protein kinases (AMPK/MAPK), phosphoinositide 3-kinase (PI3K)/AKT/mechanistic target of rapamycin (mTOR) and FAK-Src pathways^6,8,14^. It is thus evident that β-elemene may exert anti-NSCLC effects by participating in the regulation of anoikis-related pathways and that the targets of β-elemene may play key roles in the anoikis process.

Noncoding RNAs (ncRNAs) are unique RNA transcripts that are widely found in eukaryotes, and a variety of ncRNAs, including long noncoding RNAs (lncRNAs), microRNAs (miRNAs), and circular RNAs (circRNAs), are oncogenic drivers and tumour suppressors of major tumours^15^. Extensive interactions also exist between ncRNAs, with lncRNAs usually acting as specific competing endogenous RNAs (ceRNAs), competing for complementary miRNA binding sites to influence and regulate the expression of cancer target genes^16,17^. A variety of ncRNAs play key roles in NSCLC and can influence NSCLC development through various mechanisms^18–20^. In particular, anoikis-associated ncRNAs have been shown to be key markers for tumour metastasis and progression, including breast cancer^21^, hepatocellular carcinoma^22^, and prostate cancer^23^. The latest research shows that lncRNA-miRNA interactions are successfully predicted based on multiple network algorithms, providing novel and valuable insights into ncRNA prediction of prognosis of NSCLC patients^24,25^. However, studies involving the regulatory relationship of anoikis-related ncRNAs in NSCLC have been less frequently reported. Additionally, it is not yet known whether the target of β-elemene interacts with anoikis-related ncRNAs. Thus, elucidating their roles in NSCLC may improve our understanding of the mechanism of action of β-elemene in anoikis as well as new therapeutic strategies against NSCLC.

In this study, we explored the molecular expression pattern of anoikis prognostic factors in NSCLC patients by mining The Cancer Genome Atlas (TCGA) database, and investigated the biological function and prognostic significance of these molecular clusters. In addition, by constructing a lncRNA-miRNA-mRNA network of anoikis and β-elemene targets, the regulatory relationship of anoikis-associated ncRNAs on β-elemene targets was clarified. Finally, potential targets were obtained by constructing a prognostic regression model, and binding stability was evaluated for targets and β-elemene through molecular docking, providing a theoretical basis and new possibilities for the diagnosis and treatment of NSCLC.

## Methods and materials

### Data acquisition

First, the transcriptional expression profiles, clinical information, survival information, somatic mutation data, copy number variation (CNV) data, and miRNA expression profiles (isoform expression quantification) of the lung adenocarcinoma (LUAD) and lung squamous cell carcinoma (LUSC) cohorts were obtained from the TCGA database (https://portal.gdc.cancer.gov/). The TCGA-LUAD cohort included data for 59 paracancerous tissue samples and 541 tumour tissue samples, and the TCGA-LUSC cohort included data for 51 paracancerous tissue samples and 502 tumour tissue samples. Using the ComBat function of the "sva" package, the gene expression profiles of the two cohorts were combined, batch effects were eliminated, and the merged matrix was normalized using the log2 function. Only 970 NSCLC tumour samples with full transcriptome, miRNA expression profiles, and survival information were included for the construction of prognostic models. Second, 501 anoikis-associated genes (AAGs) with relevance scores greater than 0.4 were obtained from the GeneCards database^26^ (https://www.genecards.org/). In addition, 139 AAGs were obtained from the Harmonizome database^27^ (http://amp.pharm.mssm.edu/Harmonizome). A total of 638 AAGs were obtained after deduplicating. In addition, the PubChem database^28^ (https://pubchem.ncbi.nlm.nih.gov/) was explored to obtain canonical SMILES for β-elemene (PubChem CID: 6,918,391). Potential targets of β-elemene were predicted with the SwissTargetPrediction database^29^ (http://swisstargetprediction.ch/). Next, the UniProt IDs of the 23 predicted targets were converted to gene symbols using the UniProt database^30^ (https://www.uniprot.org). Finally, 26 potential target genes of β-elemene were obtained.

### Molecular characterization of AAGs

First, the expression of AAGs in paracancerous and tumour tissues of NSCLC patients was compared to obtain AAGs that were significantly differentially expressed in tumour tissues (|logFC|> 1 and *FDR* < 0.05). Then, univariate Cox regression analysis was performed on the differential AAGs to obtain the AAGs significantly associated with prognosis of NSCLC (*P* < 0.05). To further explore the genomic activity of potentially prognostic AAGs, we assessed the frequency of CNV occurrence in these AAGs and visualized the location of CNVs. In addition, to explore the interactions of prognostically relevant AAGs, we analysed the interactions of proteins encoded by AAGs using the String database^31^ (https://string-db.org/) (confidence > 0.4).

### Consensus clustering of AAGs

To explore the molecular expression patterns of prognostic AAGs, consensus clustering was performed based on the expression profiles of AAGs using the k-means algorithm of the "ConsensusClusterPlus" package^32^. The maximum number of clusters was set to 9. A resampling scheme was used to sample 80% of the sample and resampled 50 times to find stable and reliable subgroup classification. Principal component analysis (PCA) and survival analysis were applied to test the differences in clustering and to compare the expression levels of these AAGs between clusters. In addition, the correlation between AAG clusters and the clinical information of NSCLC patients was explored, and differences in biological functions between AAG clusters were compared by gene set enrichment analysis (GSEA).

### Construction and biological function analysis of the ceRNA network

To investigate the mechanism of action of β-elemene in NSCLC and its correlation with the anoikis process, we constructed an anoikis-β-elemene ceRNA network. First, anoikis-associated differential mRNAs, lncRNAs, and miRNAs (|logFC|> 0.585 and *P* < 0.05) were obtained by differential significance analysis among AAG clusters for all genes. Then, the targeting relationships of these mRNAs and lncRNAs were predicted by the DIANA-LncBase tool^33^ (http://www.microrna.gr/LncBase). The targets of differential miRNAs were also predicted by the TargetScan 8.0 database (www.targetscan.org) and compared with anoikis-related differential mRNAs and β-elemene targets. In addition, by obtaining the targeting relationships between anoikis-related miRNAs and β-elemene targets and by performing correlation analyses between anoikis-related mRNAs and β-elemene targets, anoikis-β-elemene gene relationship pairs were established for the β-elemene targets that are both miRNA targets and coexpressed genes of anoikis-related mRNAs. Finally, only mRNAs with relevance to β-elemene targets were retained, and a lncRNA-miRNA-mRNA network was established based on ncRNA-target relationship pairs. The β-elemene-regulated anoikis genes used to construct the ceRNA network were defined as β-elemene and anoikis-associated genes (BAGs). To explore the biological functions of BAGs, we analysed Gene Ontology (GO) and Kyoto Encyclopedia of Genes and Genomes (KEGG) enrichment of BAGs constructing ceRNA networks (*P* < 0.05 and *Q* < 0.1).

### Prognostic modelling of feature BAGs

To further explore the impact of BAGs on patient survival, we used the random number method to divide NSCLC patients into training and validation groups at a ratio of 1:1 and then used least absolute shrinkage and selection operator (LASSO) and random forest algorithms to screen for prognostically relevant feature BAGs. First, in the training group, we used the "randomForest" package to construct a random forest model of OS.status predictions from BAGs by randomly cycling through all possible random numbers of the variables. The number of decision trees (ntree) contained in the random forest was 500, and the number of variables used in the nodes for binary trees (mtry) was 8. The Gini coefficient method was used to determine the importance of BAGs for OS.status prediction in the random forest model, and the top 10 BAGs were taken as feature genes. In addition, a LASSO regression model was constructed using the "glmnet" package with BAGs as the independent variable and OS.status as the dependent variable in the training group. The LASSO model was used for binary discrete dependent variables (family = "binomial"), and the cross-validated loss function was expressed in terms of the mean squared error (type.measure = "deviance") with a tenfold cross-test (nfolds = 10). The number of variables corresponding to the smallest mean square error was optimal, and these BAGs were screened to be feature genes. Finally, the intersection of the feature BAGs obtained by the two algorithms was taken and the intersecting genes were used to establish a multivariate Cox regression model for predicting the prognosis of NSCLC patients. The patient's prognostic risk score was calculated using the sum of model genes and coefficient products and denoted as the BAG_Score. The training, validation, and entire groups were categorized into high and low BAG_Score groups based on the median BAG_Score of the training group. Finally, the accuracy of the prognostic model was assessed using receiver operating characteristic (ROC) curves and Kaplan‒Meier survival analyses, and multivariate Cox regression analysis was performed to assess the independent prognostic power of the BAG_Score with clinical factors.

### Construction of a clinical nomogram

A nomogram is built on the basis of multifactor regression analysis, the integration of multiple predictive indicators, and then the use of scaled line segments, according to a certain scale plotted on the same plane, to be used to express the interrelationships between the variables in the predictive model. To explore the clinical application value of the BAG_Score, we combined the BAG_Score with clinical factors (age, gender, pathologic M, pathologic N, pathologic T, tumour stage, tobacco smoking history and histological type) to construct a nomogram, assign scores according to each variable of the patient, and predict the 1-, 3-, and 5-year survival probability of the patient based on the total points. In addition, the accuracy of the nomogram for predicting OS for patient prognosis was assessed using calibration curves, cumulative risk curves, and decision curve analysis (DCA).

### Immune correlation analysis of the BAG_Score

To investigate the correlation between the BAG_Score and immune cells, we used seven algorithms, TIMER, CIBERSORT, CIBERSORT-ABS, QUANTISEQ, MCPCOUNTER, XCELL, and EPIC^34^, to assess the abundance of immune cells in patients, and the Wilcoxon test was used to compare the degree of immune infiltration with high and low BAG_Scores. In addition, patients' levels of immune escape and immune response were assessed using the Tumor Immune Dysfunction and Exclusion (TIDE) database^35^ (http://tide.dfci.harvard.edu), comparing the degree of sensitivity of the immune response in patients in the high and low BAG_Score groups.

### Mutational landscape of different BAG_Score groups

To identify the mutation profiles of patients with different PPG_scores, the "maftools" package was used to create the mutation annotation format (MAF) for the LUAD and LUSC cohorts and visualize the mutational landscapes of high and low BAG_Scores. In addition, the tumour mutational (mutation frequency per million bases) was calculated and compared between the two groups.

### Molecular docking

We obtained the protein structures of potential targets associated with β-elemene involved in building the BAG_Score model from the PDB database (https://www.rcsb.org/). Then, the 2D structure of β-elemene was obtained from the PubChem database and converted into a 3D structure using Chem3D software. Finally, the small-molecule ligand β-elemene and the potential receptor protein were molecularly docked using AutoDock Vina 1.1.2^36^ and PyMOL 2.5.5^37^, and the free energy of molecular binding was calculated to evaluate the stability of the binding.

### Statistical analysis

R software (version 4.2.1) and corresponding packages were used for statistical analysis. Cytoscape v3.9.1 was used to visualize the lncRNA-miRNA-mRNA network. Multivariate Cox regression was used to construct a prognostic model. The Kaplan‒Meier method and log-rank test were used to assess prognosis. Correlation analysis was performed using Pearson's and Spearman's methods. The Wilcoxon test was used to compare the differences between the two groups. All tests with *P* < 0.05 indicate statistical significance.

## Results

### Molecular characterization of AAGs

**Figure 1:**
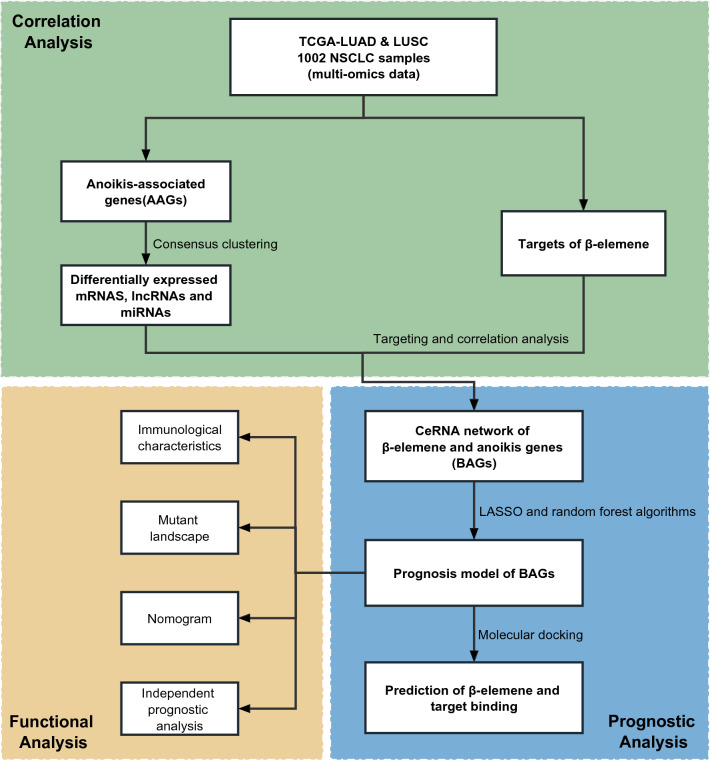
Workflow diagram.

**Figure 2:**
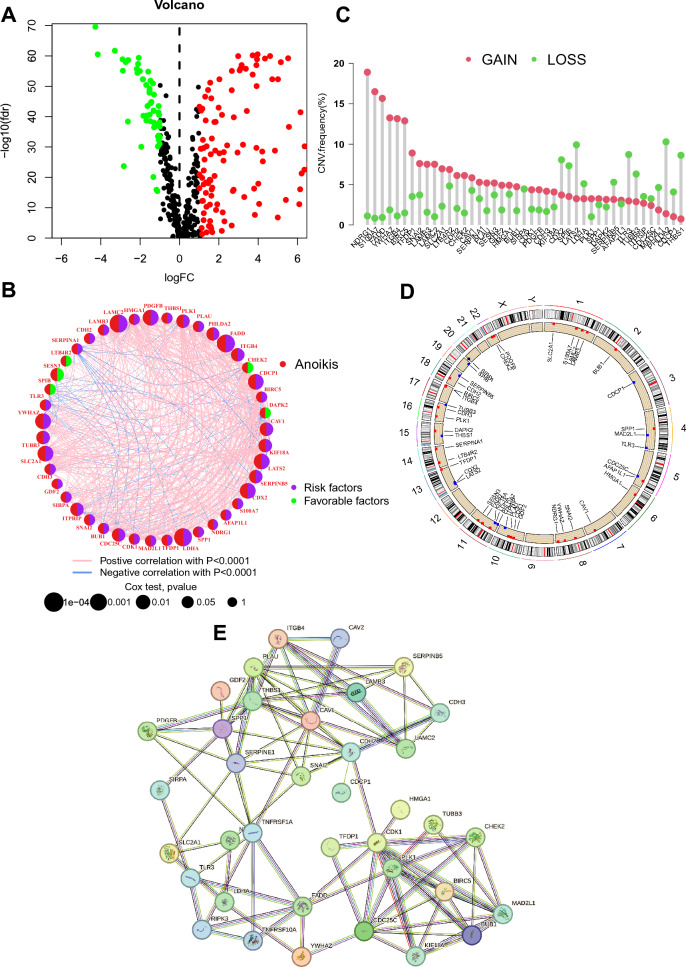
Molecular characteristics of anoikis associated genes (AAGs) (**A**) Significant difference analysis of AAGs between paracancerous (control group) and tumour tissues. (**B**) Correlation and prognosis analysis of AAGs. (**C**) Copy number variation (CNV) frequency of AAGs. (**D**) Location of CNV occurrence of AAGs. (**E**) Interaction of proteins encoded by AAGs.

The workflow diagram of the study is displayed in Fig. 1. We performed a differential significance analysis of 638 AAGs and obtained 162 AAGs that were differentially expressed in paracancerous and tumour samples of NSCLC patients (Fig. 2A). Univariate Cox regression analysis of these 162 differential AAGs yielded 43 AAGs that were significantly associated with prognosis (Fig. 2B). Among them, 38 AAGs were risk prognostic factors and most of them were positively correlated with each other (Fig. 2B). Then, by analysing the frequency of CNVs in prognosis-associated AAGs, we found that *NDRG1*, *S100A7*, and *FADD* had the highest frequency of copy number gain, whereas *THBS1*, *LATS2*, and *PHLDA2* had the highest frequency of copy number loss, and that most of the AAGs had high-frequency CNVs (Fig. 2C), which often occurred on chromosomes 1, 10, 11, etc. (Fig. 2D). In addition, Fig. 2E shows that the 37 AAGs encode proteins that have interacting relationships with each other. All these results indicate that AAGs are aberrantly expressed and genomically unstable in NSCLC tumour tissues and may contribute to the poor prognosis of patients through synergistic effects.

### Generation and functional characterization of AAG clusters

**Figure 3:**
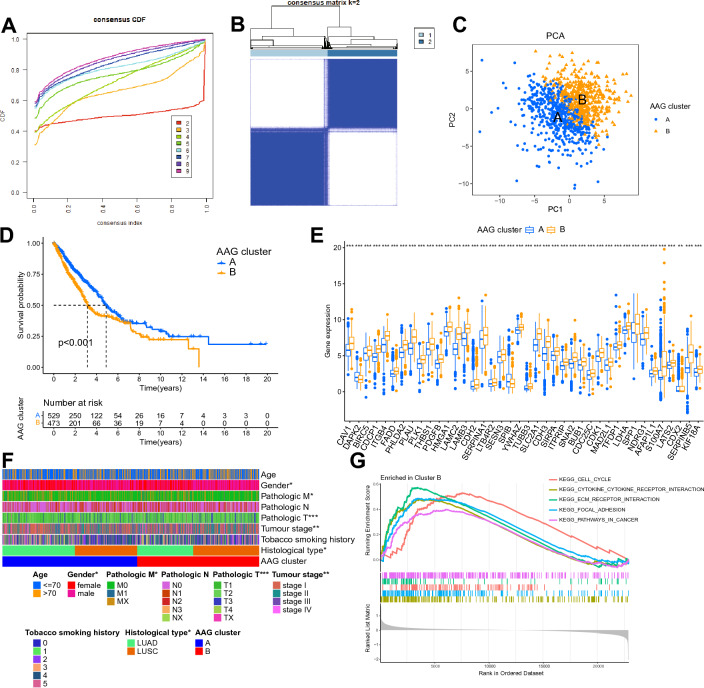
Molecular classification and function of AAGs. (**A**) Cumulative distribution function for k = 2–9. (**B**) Consensus clustering heatmap with k = 2. (**C**) PCA between two AAG clusters. (**D**) Kaplan‒Meier survival analysis between two AAG clusters. (**E**) Differential expression of AAGs between two AAG clusters. (**F**) Difference analysis of clinical characteristics between AAG clusters. (**G**) GSEA to compare two clusters.

To explore the molecular expression patterns of 43 prognostically relevant AAGs, we used consensus clustering to characterize the expression of AAGs in NSCLC. Based on the plot of the cumulative distribution function, it was determined that the clustering had optimal stability at k = 2 (Fig. 3A,B). PCA demonstrated the differential distribution of AAG cluster A and B (Fig. 3C). Kaplan‒Meier analysis illustrated that cluster A had a higher survival probability than cluster B (Fig. 3D, P < 0.001). Most AAGs were significantly highly expressed in cluster B compared to cluster A (Fig. 3E, P < 0.05). According to Fig. 3F, the clinical characteristics of gender, pathologic M, pathologic N, pathologic T, tumour stage and histological type were significantly different between the two clusters (Fig. 3F, P < 0.05). To explore the molecular functions of the differences between the two AAG clusters, we performed GSEA, which showed that compared with cluster A, cell cycle, cytokine receptor interaction, extracellular matrix (ECM)-receptor interaction, focal adhesion, and pathways in cancer were significantly enriched in cluster B (Fig. 3G). All of these results reveal that NSCLC can be clustered into two clusters with different prognostic features, clinical traits, and molecular functions based on the expression of AAGs and that AAGs may be related to the cell cycle and the pathway of tumour development in NSCLC.

### Construction of the ceRNA network of anoikis and β-elemene

**Figure 4:**
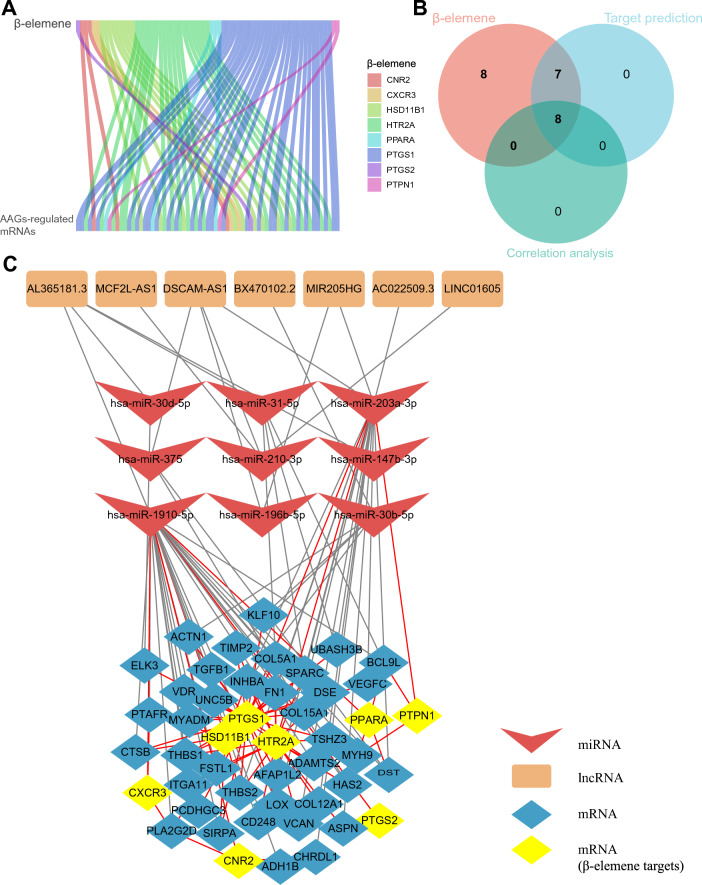
Construction of the lncRNA-miRNA-mRNA network. (**A**) Correlation analysis of β-elemene targets and AAG-regulated mRNAs. (**B**) Screening the gene sets related to anoikis among the β-elemene targets. (**C**) LncRNA-miRNA-mRNA network of the interaction between β-elemene and anoikis.

First, we performed a significant difference analysis of all the genes of AAG cluster A and B using |logFC|> 0.585 and *P* < 0.05 as thresholds, respectively, and obtained 731 mRNAs, 42 lncRNAs and 10 miRNAs significantly related to AAGs. Then, the DIANA-LncBase tool was used to compare the targeting relationship between the differential lncRNAs and miRNAs, and the relationship pairs of 9 miRNAs and 7 lncRNAs were obtained. The target genes of 9 miRNAs were predicted by TargetScan 8.0: (i) target genes of 9 miRNAs were compared with 731 differential mRNAs, and 208 mRNAs related to AAGs were obtained; (ii) 15 genes were the target genes of AAG-associated miRNAs by comparison with 26 β-elemene target genes. In addition, 15 β-elemene target genes were subjected to Spearman correlation analysis with 208 AAG-related mRNAs, and 47 genes related to β-elemene and anoikis were obtained, including 40 differential mRNAs, 8 β-elemene targets, and the intersecting gene *PTGS2* (Fig. 4A,B). Finally, based on the lncRNA-miRNA and miRNA-mRNA targeting relationships and the correlation relationship between β-elemene and anoikis, a ceRNA network consisting of 7 lncRNAs, 9 miRNAs, and 47 mRNAs was successfully constructed (Fig. 4C, Table 1). A total of 63 BAGs were obtained based on β-elemene and anoikis regulatory relationships in the network, which will help us further understand the role of β-elemene in regulating anoikis in the prognosis of NSCLC patients.

**Table 1:** ceRNA network constructed by interacting genes of β-elemene and anoikis.

Biotype	Genes
lncRNA	AL365181.3, DSCAM-AS1, MIR205HG, AC022509.3, LINC01605, MCF2L-AS1, BX470102.2
miRNA	hsa-miR-31-5p, hsa-miR-1910-5p, hsa-miR-375, hsa-miR-30b-5p, hsa-miR-30d-5p, hsa-miR-210-3p, hsa-miR-196b-5p, hsa-miR-147b-3p, hsa-miR-203a-3p
mRNA	PTPN1, PPARA, PTGS1, HSD11B1, CXCR3, HTR2A, CNR2, PTGS2, MYH9, BCL9L, DST, ACTN1, ELK3, DSE, AFAP1L2, CTSB, KLF10, COL5A1, INHBA, TGFB1, UNC5B, THBS2, LOX, TSHZ3, TIMP2, ADAMTS2, VDR, FN1, UBASH3B, PCDHGC3, SIRPA, SPARC, VEGFC, COL15A1, MYADM, PTAFR, FSTL1, THBS1, CHRDL1, ADH1B, PLA2G2D, COL12A1, CD248, VCAN, ITGA11, HAS2, ASPN

### Functional enrichment analysis of BAGs

**Figure 5:**
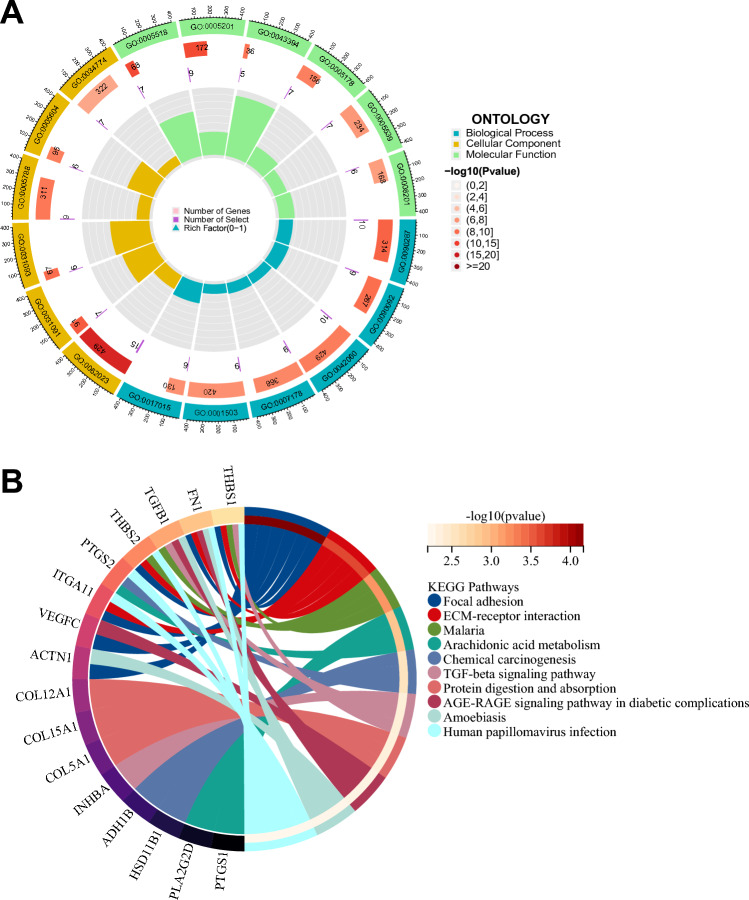
Functional enrichment of the lncRNA-miRNA-mRNA network. (**A**) GO and (**B**) KEGG analysis of interacting genes of β-elemene and anoikis in the network.

To explore the molecular functions of β-elemene and anoikis in NSCLC, we performed GO and KEGG enrichment analysis of 63 BAGs (*P* < 0.05 and *Q* < 0.1). These genes were significantly enriched in functions and pathways such as regulation of cellular response to growth factor stimulus (GO:0,090,287), epithelium migration (GO:0,090,132), tissue migration (GO:0,090,130), focal adhesion (hsa04510), ECM-receptor interaction (hsa04512), and chemical carcinogenesis (hsa05204) (Fig. 5A,B). Among them, epithelial and tissue migration are important characteristics of tumour cells; focal adhesion is the key to signal transduction; the extracellular matrix (ECM) is closely related to the immune microenvironment; and chemical carcinogens are a common driver of tumours. This indicates that BAGs may be involved in tumour characteristics, carcinogenic factors, and immune function-related functions, thus affecting the disease process of NSCLC patients.

### Prognostic BAG_Score model construction

**Figure 6:**
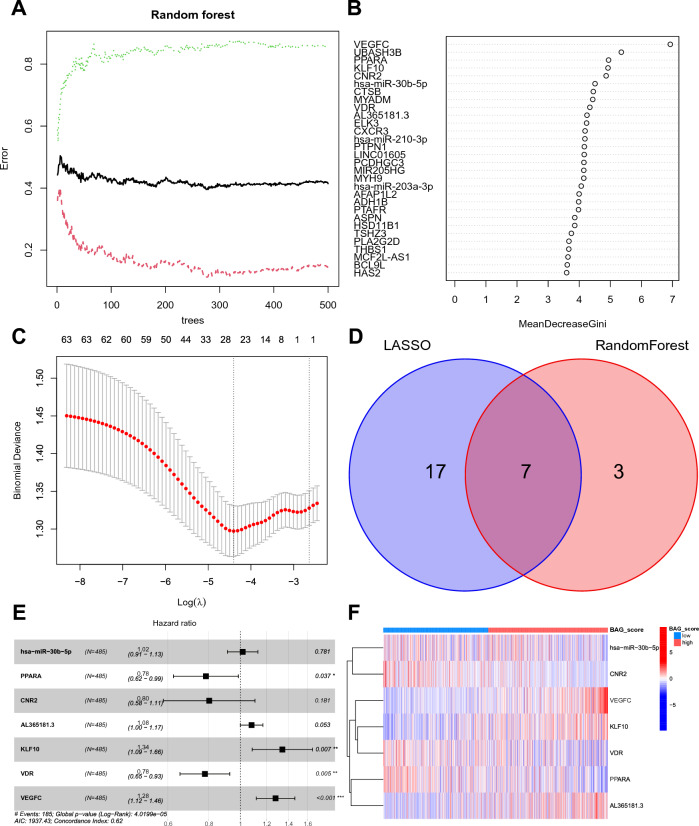
Construction of the prognostic BAG_Score model. (**A**) Model error plot for random forest. (**B**) Gene importance scoring with the random forest algorithm. (**C**) Optimal number of variables for LASSO regression analysis. (**D**) Intersecting genes of the two algorithms. (**E**) Multivariate Cox regression to construct the BAG_Score model. (**F**) Risk heatmap for BAG_Score modelling genes.

To explore the prognostic role of the β-elemene- and anoikis-regulated ceRNA network, we screened and modelled 63 BAGs. First, 970 patients were randomized into the training group (485 patients) and the validation group (485 patients). The random forest algorithm was used for the training group to construct a classifier for predicting OS.status, and according to the model error plot, the error tended to remain stable at a decision tree of 300 (Fig. 6A). The top 10 genes were obtained as feature BAGs based on the Gini coefficient method with the MeanDecreaseGini index as the importance score (Fig. 6B). Then, with OS.status as the response variable and the normalized expression matrix of the 63 BAGs as the independent variables in the training group, a tenfold cross-test was performed to obtain the smallest mean squared error (binomial deviation) of the model for a variable number of 24 (Fig. 6C). The intersection of the feature genes obtained by the two algorithms was taken to obtain seven feature BAGs for constructing the BAG_Score model (Fig. 6D). The risk score was calculated by multivariate Cox regression analysis as follows:

$$\begin{aligned} {\text{BAG}}\_{\text{Score }} = & \, \left( {0.0{15}} \right) \, *{\text{ Exp }}\left( {hsa - miR - 30b - 5p} \right) \, \\ & + \, \left( { - 0.{243}} \right) \, *{\text{ Exp }}\left( {PPARA} \right) \, + \, \left( { - 0.{22}} \right) \, *{\text{ Exp }}\left( {CNR2} \right) \, \\ & + \, \left( {0.0{78}} \right) \, *{\text{ Exp }}\left( {AL365181.3} \right) \, + \, \left( {0.{294}} \right) \, *{\text{ Exp }}\left( {KLF10} \right) \, \\ & + \, \left( { - 0.{25}} \right) \, *{\text{ Exp }}\left( {VDR} \right) \, + \, \left( {0.{247}} \right) \, *{\text{ Exp }}\left( {VEGFC} \right). \\ \end{aligned}$$

According to the BAG_Score model in Fig. 6E, *PPARA* and *VDR* were prognostic protective factors for NSCLC (*HR* < 1 and* P* < 0.05), while *KLF10* and *VEGFC* were prognostic risk factors (*HR* < 1 and *P* < 0.05). According to the risk heatmap, *CNR2*, *PPARA*, and *VDR* were relatively highly expressed in the low BAG_Score group, whereas *AL365181.3*, *KLF10*, and *VEGFC* were relatively highly expressed in the high BAG_Score group (Fig. 6F). These four genes not only are the key genes by which β-elemene regulates anoikis but also serve as potential prognostic molecular markers for NSCLC patients, providing a research basis for the study of effective targets for β-elemene.

### Assessment of prognostic BAG_Score models

**Figure 7:**
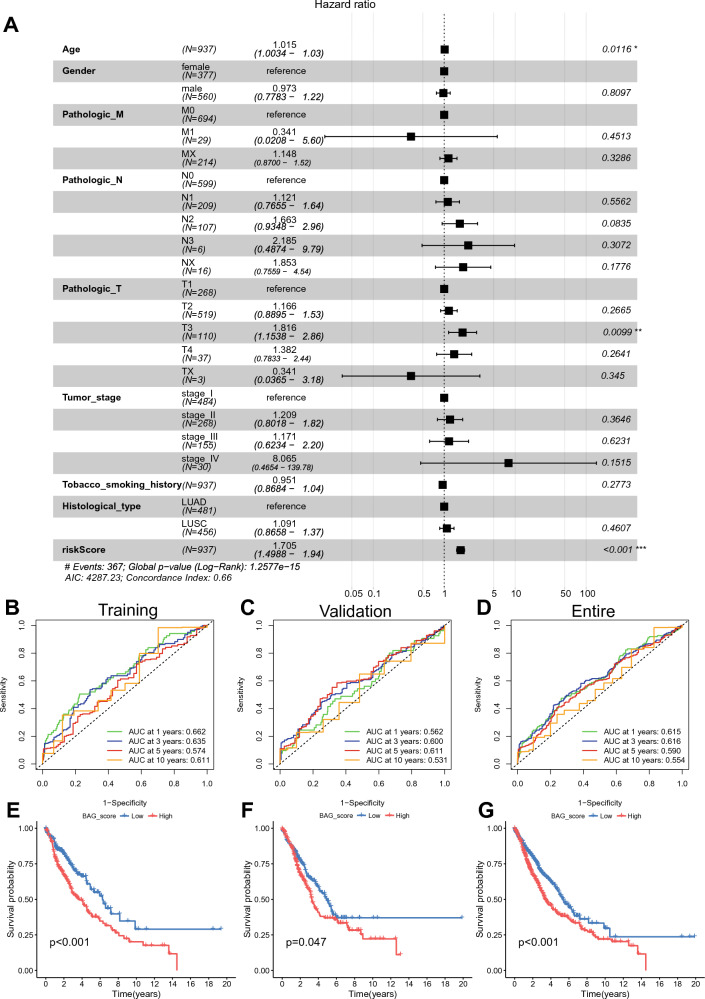
Evaluation of the BAG_Score model. (**A**) Independent prognostic analysis of BAG_Score and clinical variables. (**B**–**D**) ROC curves of the BAG_Score in the training (**B**), validation (**C**) and entire (**D**) groups. (**E**–**G**) Kaplan‒Meier survival analysis of the BAG_Score in the training (**E**), validation (**F**) and entire (**G**) groups.

Multivariate regression analysis of clinical characteristics and the BAG_Score showed that age, T3, and the BAG_Score were independent prognostic factors in NSCLC patients (Fig. 7A, *HR* > 1, *P* < 0.05). ROC analysis showed that the area under the curves (AUCs) for 1-, 3-, and 5-year survival probability were all greater than 0.57 in the training group, greater than 0.53 in the test group, and greater than 0.55 in the entire group (Fig. 7B,C,D). In particular, the AUC for 1- and 3-year survival in the entire group was greater than 0.61 (Fig. 7D), suggesting that the model has some prognostic predictive ability and is more accurate in predicting early-stage disease. Kaplan‒Meier analysis demonstrated that patients in the high and low BAG_Score groups had significantly different OS. Patients in the low BAG_Score group had a higher survival probability than those in the high BAG_Score group (Fig. 7E,F,G, log-rank test: *P* < 0.05). By evaluating the BAG_Score model, we found that the BAG_Score was an independent predictor and significantly correlated with NSCLC patient prognosis.

### Construction of the BAG_Score combined nomogram

**Figure 8:**
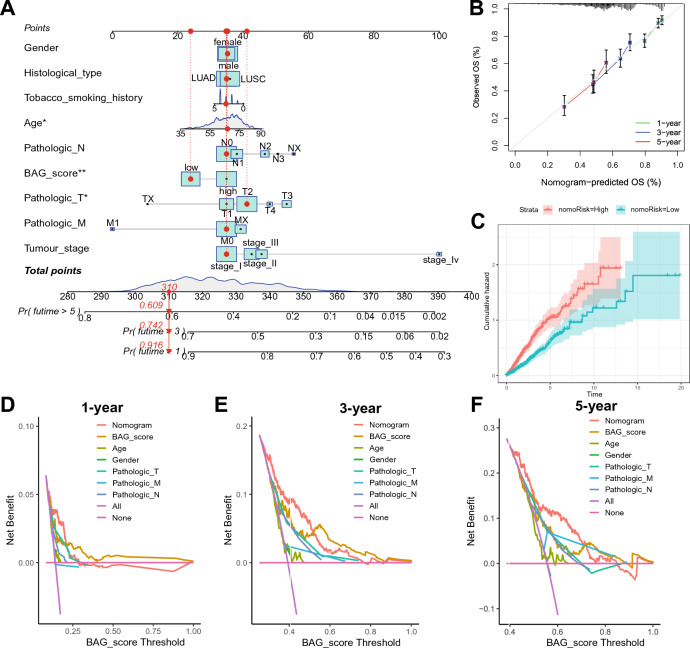
Construction of the clinical nomogram. (**A**) Construction of the nomogram for the BAG_Score and clinical characteristics. (**B**–**F**) Calibration curves (**B**), cumulative hazard curves (**C**) and 1-, 3-, and 5-year DCA curves (**D**–**F**) to assess the performance of the nomogram.

To explore the value of applying the BAG_Score with clinical factors, we constructed a clinical nomogram of the combined BAG_Score, aiming at the prediction of patient OS at 1, 3, and 5 years. In the nomogram, age, BAG_Score, and pathologic T independently predicted patient prognosis (Fig. 8A). The calibration curves show that the OS predictions for the 1-, 3-, and 5-year nomograms are extremely close to the actual observed values, demonstrating the excellent accuracy of the model (Fig. 8B). The cumulative hazard analysis showed that the cumulative hazard was higher in patients with high-nomoRisk than in the low-nomoRisk group (Fig. 8C). In addition, the 1-, 3-, and 5-year DCA curves showed that the prognostic predictive power of the BAG_Score and nomogram was superior to that of other clinical factors, resulting in a higher net clinical benefit for patients (Fig. 8D,E,F). The clinical nomogram with the BAG_Score has better predictive validity than single clinical factors and can provide an effective reference for the clinical prediction of OS in NSCLC patients.

### Immunization and mutational landscapes for different BAG_Scores

**Figure 9:**
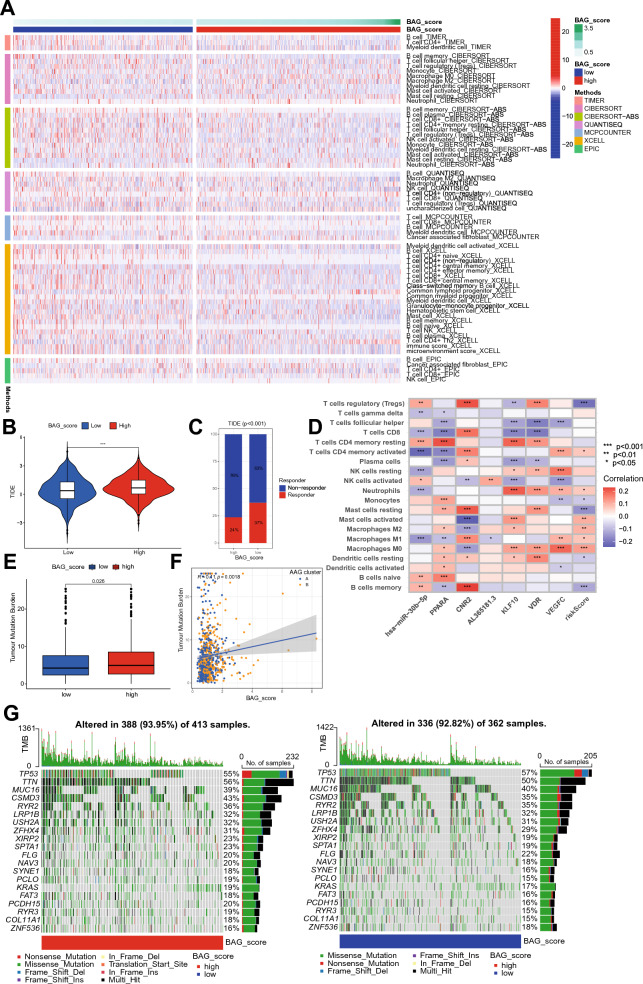
Comparison of immunization and mutational landscapes between different BAG_Score groups. (**A**) Correlation between the BAG_Score and immune cell abundance. (**B**) Differences in TIDE scores between the high and low BAG_Score groups. (**C**) Differences in the incidence of immune response between the high and low BAG_Score groups. (**D**) Correlation of BAG_Score-modelled genes and immune cell content. (**E**) Differences in TMB between the high and low BAG_Score groups. (**F**) Correlation between the BAG_Score and TMB. (**G**) Immune landscape of the high and low BAG_Score groups.

Using seven immune cell assessment algorithms to correlate the intensity of immune cell infiltration and the BAG_Score in NSCLC patients, the abundances of a total of 65 kinds of immune cells were correlated with the BAG_Score. Most of the immune cell levels were high in the low BAG_Score group, such as CD4 + T cell central memory, CD4 + T cell effector memory, and CD8 + T cell (Fig. 9A) levels, suggesting that the immune cells were more active in patients with low BAG_Scores. The low BAG_Scores group had significantly lower TIDE scores than the high BAG_Score group, suggesting that the low BAG_Score group was less prone to immune escape (Fig. 9B). Moreover, the incidence of immune response reactions was significantly higher in the low BAG_Score group than in the high BAG_Score group (Fig. 9C). Among the modelling genes, *CNR2* and *PPARA* were significantly correlated with more immune cells, CD8 T cells and follicular helper T cells were negatively correlated with the modelling genes, and neutrophils and M0 macrophages were positively correlated with the modelling genes (Fig. 9D). This indicates that the BAG_Score is closely related to the immune level of NSCLC patients and that patients with a low BAG_Score have stronger immune cell activity and a higher success rate of immune response occurrence.

To explore the relationship between the BAG_Score and genomic instability, we assessed and compared the differences in tumour mutation burden (TMB) between the high and low BAG_Score groups. The outcomes revealed that TMB was significantly higher in the high BAG_Score group than in the low BAG_Score group (Fig. 9E, P < 0.05). Moreover, the BAG_Score was significantly positively correlated with TMB (*COR* = 0.11, *P* = 0.0018), and AAG cluster A had a lower TMB and BAG_Score (Fig. 9F). Furthermore, visualizing the immune landscape of the high and low BAG_Score groups in Fig. 9G, gene alterations occurred more frequently in the high BAG_Score group (93.95%) than in the low BAG_Score group (92.82%). The most frequent mutation type in both groups was missense mutation. The most mutation-prone genes in both groups were *TP53*, *TTN*, *MUC16*, *CSMD3* and *RYR2*. Except for *TP53*, all of these genes were mutated more frequently in the high BAG_Score group than in the low BAG_Score group (Fig. 9G). In summary, it can be concluded that patients in the high BAG_Score group have a higher probability of mutations and greater genomic instability, which may lead to faster tumour progression and worse prognosis.

### Molecular docking of β-elemene

**Figure 10:**
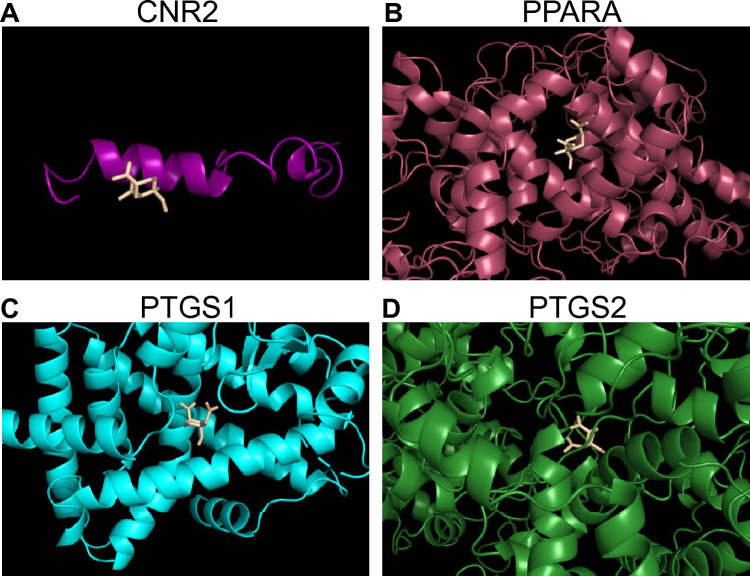
Molecular docking. Purple (**A**), red (**B**), blue (**C**) and green (**D**) represent the protein structures of CNR2, PPARA, PTGS1 and PTGS2, respectively. The wheat-coloured structure represents β-elemene.

According to the BAG_Score model, *CNR2* and *PPARA* are potential targets of β-elemene; *KLF10*, *VDR* and *VEGFC* all have a coexpression relationship with the β-elemene target *PTGS1*; *PTGS2* and *PTGS1* belong to the same gene family and are also potential targets of β-elemene; and in particular, *PTGS2* is also a gene related to anoikis. Therefore, we selected *CNR2*, *PPARA*, *PTGS1* and *PTGS2*, four key genes that have direct or indirect effects on prognosis in NSCLC, and conducted molecular docking with β-elemene. The molecular docking effect of these four macromolecule receptors and the small-molecule ligand β-elemene was evaluated through AutoDock Vina 1.1.2. The results showed that the four proteins all have the ability to bind to β-elemene (Fig. 10A,B,C,D). Among them, the combination of *PPARA* and β-elemene was the most stable (binding free energy − 6.0 kcal/mol), followed by *PTGS2* (− 5.8 kcal/mol) and *PTGS1* (− 5.2 kcal/mol), and *CNR2* was the least stable (− 4.4 kcal/mol). This suggests that the four proteins all have strong affinity for β-elemene and may be potential prognostic targets for β-elemene in NSCLC patients.

## Discussion

NSCLC is a serious global public health problem, and despite ongoing advances in diagnostic and therapeutic technologies, NSCLC remains an incurable disease for most patients^38^. Curcumae Rhizoma has a long history of use in TCM clinical NSCLC treatment, and modern studies have demonstrated that Curcumae Rhizoma exerts anti-NSCLC effects via multiple pathways through a variety of active constituents, among which β-elemene is the most important sesquiterpene compound isolated from Curcumae Rhizoma and is rich in prominent antitumour activity^39,40^. Therefore, the continuous elucidation of the β-elemene antitumour mechanism is of increasing significance for exploring the innovative path of antitumour drug development.

In this study, we characterized the genetic and epigenetic profiles of anoikis in NSCLC patients by gene expression and clinical information from the LUSC and LUAD cohorts of the TCGA database. Anoikis genes have significant prognostic differences and genomic instability, suggesting that AAGs are closely associated with tumour development. To explore the molecular mechanisms regulating anoikis in NSCLC patients, we used consensus clustering to analyse the molecular expression patterns of patients. According to the AAG expression level, patients can be classified into two different AAG molecular subtypes, and NSCLC patients with different clusters have different prognoses, clinical features and immune functions.

Then, to further explore the molecular regulatory mechanism of AAGs on tumour cells, we successfully constructed a lncRNA-miRNA-mRNA regulatory network of β-elemene interaction with anoikis by using the targeting relationship of AAG-related lncRNAs, miRNAs and mRNAs as well as through the interactions between β-elemene and AAG-related genes. This ceRNA network demonstrated the rich interactions between β-elemene targets and related genes of anoikis in NSCLC, which provided a new theoretical basis and direction to further explore the antitumour mechanism of β-elemene in NSCLC.

In addition, to further explore the prognostic role of β-elemene in NSCLC, we screened and constructed a prognostic score model for all the genes in the ceRNA network using LASSO regression and a random forest algorithm. The scoring model can be used as an independent prognostic factor for the prediction of prognosis in NSCLC patients and in conjunction with clinical factors to construct a nomogram that provides the best net benefit for the prediction of survival in clinical patients. Molecular docking of β-elemene and model-related targets is beneficial for elucidating the mechanism of β-elemene in NSCLC patients.

In the prognostic scoring model, we identified Peroxisome proliferator activated receptor alpha (*PPARA*) and Cannabinoid receptor 2 (*CNR2*) as potential target genes of β-elemene, which we hypothesized could be regulated by upstream *hsa-miR-1910-5p*. *PPARA* was the first PPAR isoform identified, and several studies have demonstrated the oncogenic effects of *PPARA* in NSCLC: *PPARA* activation inhibits NSCLC growth and angiogenesis and reduces metastasis^41^; the *PPARA* agonist fenofibrate promotes NSCLC resistance to gefitinib by modulating in a *PPARA*-dependent manner the AMPK/AKT/forkhead box-1 (FOXO1) pathway to promote gefitinib-induced apoptosis, thereby alleviating NSCLC resistance to gefitinib^42^; N-Acetyl-Cysteine (NAC) in combination with *PPARA* induces *p53* and reduces p65 protein expression to inhibit 3-phosphoinositide-dependent protein kinase 1 (PDK1) activity, leading to inhibition of NSCLC cell growth^43^. *CNR2* is widely present in NSCLC tissues, and its expression influences NSCLC development. Xu et al.^44^ found that deletion of *CNR2* inhibited the progression of NSCLC cells, suggesting that *CNR2* has a pro-carcinogenic role in NSCLC. Sarsembayeva et al.^45^ demonstrated that *CNR2* in the tumour microenvironment hinders the antitumour activity of CD8 T cells and NK cells, thereby promoting NSCLC growth. It has been shown that *CNR2* expression in early-stage NSCLC is associated with prolonged survival and fewer lymph node metastases^46^. Both *PPARA* and *CNR2*, potential target genes of β-elemene, have been shown to be involved in the tumour development process of NSCLC. Among them, *PPARA* is an oncogene, which is consistent with our observation, so we speculate that β-elemene exerts tumour suppression in NSCLC through the oncogene *PPARA*.

Both KLF transcription factor 10 (*KLF10)* and *hsa-miR-30b-5p* were modelled, and interestingly, *KLF10* is a potential target gene for *hsa-miR-30b-5p*. We hypothesized that *hsa-miR-30b-5p* might improve the prognosis of patients by targeting *KLF10*. Studies have shown that *miR-30b-5p* is closely associated with the survival and prognosis of lung cancer patients^47,48^. Qiu et al.^49^ found that *miR-30b-5p* plays an oncogenic role in lung cancer and sensitizes lung cancer cells to cisplatin by targeting low-density lipoprotein receptor-related protein-8 (LRP8). It has been demonstrated that *miR-30b* can induce anoikis resistance by downregulating Caspase 3 expression^50^.

We found that the anoikis-associated lncRNA *AL365181.3* may be regulated by hsa-*miR-31-5p* to exert pro-oncogenic effects. In previous studies, *AL365181.3* was identified as a neutrophil extracellular trap-associated lncRNA and has potential prognostic value for LUAD patients^51^. In addition, *AL365181.3* has been identified as an iron death-associated lncRNA and has been verified to be significantly upregulated in a variety of NSCLC cell^52^. Yu et al.^53^ found that *miR-31-5p* was heavily enriched in the exosomes of hypoxic LUAD cells and promoted LUAD invasion and migration by decreasing special AT-rich sequence-binding protein 2 (SATB2) expression and activating MEK/ERK signalling pathway transduction. Zhu et al.^54^ demonstrated that *miR-31-5p* is highly expressed in LUAD and determined that it promotes LUAD progression through the tensin1 (*TNS1)*/*p53* axis. Zhu et al.^55^ demonstrated that *miR-31-5P* targeting the hypoxia inducible factor 1α inhibitor/hypoxia inducible factor (FIH/HIF) mechanism enhances the Warburg effect, induces glycolysis and promotes NSCLC cell proliferation. Thus, both *hsa-miR-31-5p* and *AL365181.3* are upregulated in NSCLC, leading to anoikis inhibition and exerting a potential pro-oncogenic role.

In addition, both vitamin D receptor (*VDR*) and vascular endothelial growth factor C (*VEGFC*) were predictive of NSCLC prognosis, and both were coexpressed with the target gene β-elemene and the target gene prostaglandin-endoperoxide synthase 1 (*PTGS1*). A high expression level of *VDR* is associated with elevated LUAD survival and with anti-proliferative and G1 arrest^56^. Several studies have shown that *VDR* polymorphisms can significantly reduce NSCLC risk^57,58^. High expression of *VEGFC* is significantly associated with poor prognosis in NSCLC. Qin et al.^59^ found that coexpression of *VEGFC* and programmed cell death ligand 1 (PD-L1) was an indicator of high risk of recurrence and poor prognosis in LUAD. Regan et al.^60^ demonstrated that *VEGFC* is a major driver of tumour lymphangiogenesis in NSCLC. *PTGS1* is a coexpressed gene of *VDR* and *VEGFC* in NSCLC, while homologous prostaglandin-endoperoxide synthase 2 (*PTGS2)* is one of the target genes of β-elemene. *PTGS1* and *PTGS2* are important anticancer targets, and studies have shown that *PTGS1* and *PTGS2* are highly associated with lung tumorigenesis^61,62^. Several studies have demonstrated the association of *PTGS2* with anoikis: *PTGS2*-mediated prostaglandin E2 (PGE2) synthesis renders three-dimensionally cultured mesenchymal stem cells (MSCs) resistant to anoikis^63^; *PTGS2* provides hepatocyte growth factor-mediated resistance to anoikis and promotes human head and neck squamous cell carcinoma growth^64^; and a *PTGS2* inhibitor (celecoxib) enhances the effect of cisplatin and induces anoikis in osteosarcoma through the PI3K/Akt pathway^65^. For β-elemene, Su et al.^66^ found that β-elemene combined with 5-fluorouracil (5-FU) inhibited triple-negative breast cancer growth by interfering with the nuclear factor-kappaB (NF-κB)/*PTGS2* pathway. Cai et al.^67^ found that β-elemene inhibited *PTGS2* expression in NSCLC. From this, we hypothesize that the active substance β-elemene regulates the expression of the potential molecular markers *VDR* and *VEGFC* by acting on *PTGS1* and *PTGS2* to affect anoikis in NSCLC cells.

In summary, by studying the interaction relationship between β-elemene targets and anoikis-related genes, this study obtained multiple gene pairs related to the prognosis of NSCLC patients, which provides new research ideas and a theoretical basis for the antitumour mechanism and clinical treatment of β-elemene-acting anoikis.

This study also has some limitations. External validation of the prognostic scoring model with other cohorts was not performed because of limited miRNA-seq data. Moreover, our data were obtained from public clinical databases, and relevant molecular and animal experiments are yet to be carried out to validate the interaction between β-elemene targets and genes related to loss of anoikis. At the same time, this article has certain shortcomings in methodology. The prognostic prediction feature used in this article is the expression level of gene pairs, and the existing research on the interaction between ncRNAs is very mature^24,25^. Single-cell multiomic data and related algorithms have also been widely used in gene/protein association analysis^68,69^; in addition, the potential association between metabolites and diseases can be directly predicted based on deep learning models^70–72^, but this article has not yet conducted an in-depth study of diseases based on metabolites. In the future, this research should be based on the theoretical model of the gene/protein signalling network^73–75^, and use deep learning model theory to connect with clinical practice to gain an in-depth understanding of the regulatory mechanism of the disease and find potential therapeutic targets.

## Conclusion

We comprehensively analysed the genetic and clinical characteristics and prognosis of AAGs and, for the first time, discovered a rich interaction relationship between β-elemene and AAG-regulated genes in NSCLC patients. Furthermore, we observed the effect of interacting gene pairs on prognosis, which provided us with enlightening significance and a new perspective to further explore the mechanism of action of β-elemene in NSCLC patients.

## Data Availability

All of the data used in this investigation was obtained from public clinical databases. The data set numbers are mentioned in the publication. All dataset numbers are mentioned in the article and are publicly available. All data analysis in this study is based on the R software (version 4.2.1). The methods are all from the published R package, and the specific methods can be found in the text and references. Further inquiries can be directed to the corresponding author.

## Abbreviations

NSCLC: Non-small cell lung cancer
TCM: Traditional Chinese medicine
CHM: Chinese herbal medicine
ZIC2: Zic family member 2
Src: Steroid receptor coactivator
FAK: Focal adhesion kinase
PTEN: Phosphatase and tensin homolog
AKT: Protein kinase B
FAM188B: Family with sequence similarity 188 member B
EGFR: Epidermal growth factor receptor
AMPK: The AMP-activated protein kinase
MAPK: Mitogen-activated protein kinases
PI3K: Phosphoinositide 3-kinase
mTOR: Mechanistic target of rapamycin
ncRNA: Noncoding RNA
lncRNA: Long noncoding RNA
miRNA: MicroRNA
circRNA: Circular RNA
ceRNA: Competing endogenous RNA
TCGA: The Cancer Genome Atlas
CNV: Copy number variation
LUAD: Lung adenocarcinoma
LUSC: Lung squamous cell carcinoma
AAG: Anoikis-associated gene
PCA: Principal component analysis
GSEA: Gene set enrichment analysis
BAG: β-Elemene and anoikis-associated genes
GO: Gene Ontology
KEGG: Kyoto Encyclopedia of Genes and Genomes
LASSO: Least absolute shrinkage and selection operator
ROC: Receiver operating characteristic
DCA: Decision curve analysis
TIDE: Tumor Immune Dysfunction and Exclusion
MAF: Mutation annotation format
ECM: Extracellular matrix
AUC: Area under the curves
TMB: Tumour mutation burden
PPARA: Peroxisome proliferator activated receptor alpha
CNR2: Cannabinoid receptor 2
FOXO1: Forkhead box-1
NAC: Acetyl-Cysteine
PDK1: 3-Phosphoinositide-dependent protein kinase 1
KLF10: KLF transcription factor 10
LRP8: Low-density lipoprotein receptor-related protein-8
SATB2: Special AT-rich sequence-binding protein 2
TNS1: Tensin1
FIH: Hypoxia inducible factor 1α inhibitor
HIF: Hypoxia inducible factor
VDR: Vitamin D receptor
VEGFC: Vascular endothelial growth factor C
PTGS1: Prostaglandin-endoperoxide synthase 1
PD-L1: Programmed cell death ligand 1
PTGS2: Prostaglandin-endoperoxide synthase 2
PGE2: Prostaglandin E2
MSCs: Mesenchymal stem cells
5-FU: 5-Fluorouracil
NF-κB: Nuclear factor-kappaB
